# Prevalence study of Bovine viral diarrhea virus by evaluation of antigen capture ELISA and RT-PCR assay in Bovine, Ovine, Caprine, Buffalo and Camel aborted fetuses in Iran

**DOI:** 10.1186/2191-0855-1-32

**Published:** 2011-10-21

**Authors:** Farhad Safarpoor Dehkordi

**Affiliations:** 1 Young Researchers Club, Faculty of Veterinary Medcine, Islamic Azad University, Shahrekord Branch, Shahrekord, Iran

**Keywords:** Prevalence study, Bovine viral diarrhea virus, antigen capture ELISA, RT-PCR, aborted fetuses, Iran

## Abstract

Bovine viral diarrhea virus is a pestivirus in the family Flaviviridae that cause abortions and stillbirths in livestock and its traditional diagnosis is based on cell culture and virus neutralization test. In this study, for more sensitive, specific detection and determined the prevalence of virus in aborted Bovine, Ovine, Caprine, Buffalo and Camel fetuses the antigen capture ELISA and RT-PCR were recommended. From the total of 2173 aborted fetuses, 347 (15.96%) and 402 (18.49%) were positive for presence of Bovine viral diarrhea virus by antigen capture ELISA and RT-PCR respectively. Statistical analysis of data showed significant differences between ELISA and RT-PCR for detection of virus in aborted fetuses.

These results indicate a high presence of this pathogen in Iran and that RT- PCR is considerably faster and more accurate than ELISA for identification of Bovine viral diarrhea virus.

To our knowledge the Camels and Bovine are the most resistant and sensitive to Bovine viral diarrhea's abortions respectively and the prevalence of virus in Caprine is more than Ovine aborted fetuses. This study is the first prevalence report of Bovine viral diarrhea virus in aborted Bovine, Ovine, Caprine, Buffalo and Camel fetuses by evaluation of ELISA and RT-PCR in Iran.

## Introduction

Bovine viral diarrhea virus (BVDV) is a pestivirus in the family Flaviviridae and is closely related to classical swine fever and ovine Border disease viruses (Donis 1995) that cause Bovine Viral Diarrhea (BVD) in mammals including Antilocapridae, Bovidae, Camelidae, Cervidae, Giraffidae, Suidae, Tragulidae families and small ruminants (Grondahl et al. 2003; Nettleton 1990; Van Campen et al. 2001; Loken et al. 1995). Bovine viral diarrhea virus (BVDV) has world-wide distribution and causes various clinical syndromes in cattle including diarrhea, mucosal disease, reproduction disfunctions (abortion, teratogenesis, embryonic resorption, fetal mummification and stillbirth) and hemorrhagic syndrome (Coetzer and Tustin 2004; Passler et al. 2007; Baker 1995).

The immunotolerant cattle can shed the virus via secretion and excretion into the environment for a long time, thus being a major source of BVDV infections in herds (baker 1987). Therefore, BVD with these economic losses, need to accurate and sensitive diagnostic methods for rapid identification and elimination of persistent carriers in the herds.

Confirmation that an abortion is caused by BVDV is often difficult to establish (Ruth 1987). In past, the most common method was an isolation of virus in cell cultures but it was difficult, time consuming and lengthy process that requires experienced technicians.

The serological methods such as Enzyme-linked immunosorbent assay (ELISA) usually employed for diagnosis BVDV in clinical samples (Entrican et al. 1995) but at these years several molecular methods like reverse transcriptase polymerase chain reaction (RT-PCR) are use extremely to detection of BVD viral RNA for diagnostic purposes (Kim and Dobovi 2003; Gilbert et al. 1999).

The two fold purpose of this current study were to detection of BVDV by evaluation of antigen capture ELISA and RT-PCR assay in aborted fetuses and determined the prevalence rate of this virus in aborted Bovine, Ovine, Caprine, Buffalo and Camel fetuses samples in Iran. The positive aborted fetuses for BVD were studied for presents of gross lesion.

## Materials and methods

### Samples

A total of 2173 aborted fetuses including 620 Bovine, 525 Ovine, 442 Caprine, 372 Buffalo and 214 Camel were collected from 1012 commercial herds of various area of Iran in autumn of 2010 (Table 1). All samples had only abomasal contents of aborted fetuses those collected in strile conditions and sent under refrigeration to the labrotary as soon as possible. Samples were stored at -20°C until processing.

**Table 1:** The numbers of abomasal contents of aborted Bovine, Ovine, Caprine, Buffalo and Camel fetus samples from four provinces that is located in various parts of Iran

Provinces	No. of samples				
	
	Bovine	Ovine	Caprine	Buffalo	Camel
Tehran	274	100	53	165	33

Isfahan	188	76	148	70	81

Kerman	83	152	102	25	70

Khorasan	75	197	139	112	30

Total	620	525	442	372	214

Samples were collected randomly in autumn of 2010.

### Antigen capture ELISA

All samples were tested using commercial BVDV Antigen Test Kit/Serum Plus (HerdChek, IDEXX Laboratories, Westbrook, ME, USA), in which microtitre plates were coated with anti- Erns monoclonal antibodies. The kit is based on the detection of the Erns (gp44-48) glycoprotein of the BVD virus. The abomasal content samples were diluted (1:1) by wash solution. Fifty μl of sample was loaded into wells and incubated for 2 hours at 37°C. The reaction was terminated by the addition of 100 μl of stop solution to each well and finally the absorbance at 450 nm was monitored in ELISA reader (BIO-TEK Instruments, Inc. Winooski, VT, USA). The result could be read visually where the OD was measured at450 nm. Positive and negative control were used as indicated in the kit.

The presence or absence of BVDV antigen in the sample is determined by the corrected OD value (S-N) for each sample was considered as follow:

$$\text{S}-\text{N}=\text{Samples A45}0-\text{NC}{\text{x}}^{-}$$

Samples with S-N values less or equal to 0.3 were classified as negative and samples with S-N values higher than 0.3 were classified as positive for BVDV antigen. In this study the NCx¯ is negative control.

### RNA extraction

RNA purification was performed using the RNX™_ Plus Kit (Sinagen, Iran) according to the manufacturer's instructions. Briefly 100-150 μl of viral suspension (abomasal contents and water control) were mixed with 1 ml of RNX and left for at least 5 min at 4°C. After the addition of 200 μl chloroform and mixing, the liquid was clarified by centrifugation at 12,000 × g for 15 min at 4°C. The supernatant was transferred to a new tube and mixed with an equal volume of isopropanol followed by centrifugation at 12,000 g for 15 min at 4°C. The pellet was washed with 1 ml of 70% ethanol. Finally, RNA was eluted in 50 μl of 1 mM RNase free EDTA.

### RT-PCR assay

The forward primer sequence is 5'- CAT- GCC-CCT-AGT-AGG-ACT-AGC-3', and the reverse primer sequence is 5'- TCA-ACT-CCA-TGT-GCC-ATG-TAC-3', which is use for BVDV screen in abomasal contents of aborted fetuses. All oligonucleotide primers were obtained from a commercial source (Cinna Gen, Iran). Total RNA (3 μl) was mixed with 1.5 μl ml of reverse primer (10 μM/μl) and incubated at 70°C for 5 min followed by chilling on ice. The rest of the reaction mixture contained 4 μl of 5 × first strand buffer, 2 μl of dNTPs (10 mM), 20 U of RNasin (20 U/μl), 200 U (200 U/μl) of Moloney Murine Leukemia Virus (M-MuLV) (Fermentas) and 7.5 μl d-H20 was added, followed by an incubation at 42°C for 60 min cDNA synthesis was terminated by incubation at 70°C for 10 min. PCR was performed in a 25 μl reaction mix. The final concentration of the reagent was as follows: PCR buffer (1 × time) (Cinagen, Iran), dNTP 0.2 mM), MgCl2 (1.5 mM), each primer (0.5 μM), Taq DNA polymerase (0.625). Reactions were performed in an automated thermal cycler (Bio-Rad gradient Thermal Cycle). Cycle parameters for PCR were as follows: one cycle at 95°C for 5 min followed by thirthy five cycles in 3 continous phases including 94°C for 30 sec, 55°C for 100 sec, and 72°C for 2 min, and finally terminated by a single cycle of a final extention at 72°C for 10 min.

### Gel electrophoresis

The RT-PCR-amplified products were examined by electrophoresis in a 2% agarose gel, stained with a 1% solution of ethidium bromide, and examined under UV illumination. In this study, A negative control (sterile water), and a positive control RNA from BVDV (Cinna Gen, Iran), were included in each amplification run.

### Statistical analysis

Data were transferred to Microsoft Excel spreadsheet (Microsoft Corp., Redmond, WA, USA) for analysis. Using SPSS 18.0 statistical software (SPSS Inc., Chicago, IL, USA), ANOVA test analysis were performed and differences were considered significant at values of P < 0.05.

## Results

In this study a total of 2173 abomasal content samples of aborted fetuses including 620 Bovine, 525 Ovine, 442 Caprine, 372 Buffalo and 214 Camel from 1012 commercial herds of various area of Iran were tested for presence of BVDV by evaluation of antigen capture ELISA and RT-PCR assay.

Data from antigen capture ELISA were studied and quality of RNA extracted after agarose gel electrophoresis from abomasal contents of aborted fetuses were observed and all were accepted and diagnosed suitable for RT-PCR assay.

Results indicated that from a total of 2173 abomasal content of aborted fetus samples, 347 (15.96%) and 402 (18.49%) were positive for presence of BVDV by antigen capture ELISA and RT-PCR assay respectively. Serological and molecular results indicate that the frequency of BVDV in aborted Bovine, Ovine, Caprine, Buffalo and Camel fetuses are shown in table 2.

**Table 2:** Frequency of Bovine viral diarrhea virus in aborted bovine, ovine, caprine, buffalo and camel fetuses by evaluation of antigen capture ELISA and RT-PCR methods in Iran

Species	No. of samples	Antigen capture ELISA (%)	RT-PCR (%)
Bovine	620	111 (17.90)	127 (20.48)

Ovine	525	74 (14.09)	74 (16.74)

Caprine	442	71 (16.06)	93 (17.71)

Buffalo	372	63 (16.93)	76 (20.43)

Camel	214	27 (12.61)	32 (14.95)

Total	2173	347 (15.96)	402 (18.49)

Table show that the RT-PCR method has a higher accuracy than antigen capture ELISA to detection of BVD virus.

Statistical analysis of data showed significant differences about P < 0.01 between antigen capture ELISA and RT-PCR for detection of BVDV in abomasal contents of aborted Bovine, Ovine, Buffalo and Camel fetuses and P < 0.05 between ELISA and RT-PCR for detection of BVDV in abomasal contents of aborted Caprine fetuses.

In additional to above, results showed significant differences about P < 0.01 between presence of BVDV in Camel aborted fetuses with Bovine and Buffalo and P < 0.05 between presence of BVDV in Bovine with Ovine and Caprine aborted fetuses, by using each of two diagnostic methods. The observed difference between aborted fetuses and control group is significant (P < 0.05). The S-N values that were obtained after antigen capture ELISA have significant differences about P < 0.05 between infected and non-infected aborted fetuses by BVDV.

Therefore, we can conclude that the abortion is a direct consequence of BVDV infection in the herds studied.

This present study showed that the RT-PCR is more accurate than antigen capture ELISA to detection of BVDV in aborted fetuses. Therefore, the prevalence rate of BVDV in aborted fetuses of Iran was estimated 18.49% (402 positive from 2173 samples).

Using from the negative and positive controls in all RT-PCR testing that were performed in this study, cause to all of the samples that were reported positive for presences of BVDV, had a sufficient accuracy and practical diagnostic value.

**Figure 1:**
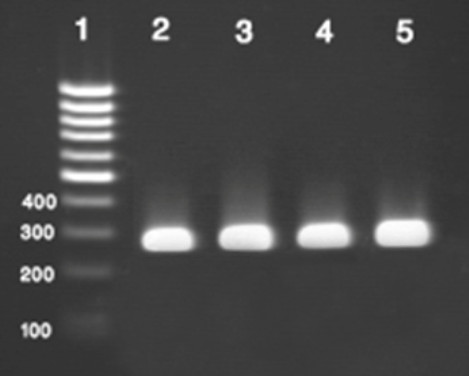
**Electrophoresis of PCR products of BVDV genome obtained by RT-PCR on 2% agarose gel electrophoresis**. 1 is 100 bp ladder, 2-4 is positive sample and 5 is positive control. In this study the length of RT-PCR product was 290 bp.

Figeres show a typical example of RT-PCR results for representative isolates of BVDV (Figure 1).

Our results showed that BVDV infection present widely in aborted Bocine, Ovine, Caprine, Buffalo and camel fetuses in the herds of Iran. The Camel is the most resistant and the Bovine is the most sensitive to BVDV's abortion.

Our results showed that the prevalence of BVDV in Caprine is more than Ovine aborted fetuses.

## Discussion

There are many factors those causes abortion in livestock but the causes of animal abortion are unknown in more than 50% of the cases. BVD is one of the most important infectious diseases which associated with infertility and abortion. Placental changes produced by BVDV could allow other pathogenic organisms to cross the fetal placenta barrier (Murray 1991).

BVD cause significant economic losses in the livestock industry in Iran. The prevalence of BVD in Iran has been mainly reported on the basis of the detection of antibody against BVDV. In early investigations a range of 20-90% of BVD incidence has been reported (Mirshamsy et al. 1970). In a study on slaughtered cattle in Tehran province, 58.51% of animals were found to be seropositive (Kargar et al. 1995). In a later survey of the cattle population in Iran, 3000 serum samples were tested and results showed that about 39.6% of young animals were seropositive. The prevalence of antibody and the proportion of seropositive animals rose markedly with age up to 62% (Sedighi-nejad 1996). Therefore, following control programs in Iran, the prevalence of BVD from 20-90% in 1970 (Mirshamsy et al. 1970) and 62% in 1996 (Sedighi-nejad 1996), decrease to 18.49% in 2011 (this present study).

In the majority of cases, confirmation that an abortion is caused by BVDV is difficult to establish (Ruth 1987) but this present study introduced antigen capture ELISA and RT-PCR assay as an accurate and sensitive diagnostic methods that can detect BVDV virus in abomasal contents of aborted fetuses. Our results indicated that the molecular method was more accurate than antigen capture ELISA that was similar to previous study (Horner et al. 1995).

Several methods for ELISA for antigen detection have been published (Vanderheijden et al. 1993; Cornish et al. 2005; Entrican et al. 1995) and a number of commercial kits are available. Most are based on the sandwich ELISA principle, with a capture antibody bound to the solid phase, and a detector antibody conjugated to a signal system, such as peroxidase. The new generation of antigen-capture ELISAs (ERNS capture ELISAs) is able to detect BVD antigen in blood as well as in plasma or serum samples. Our antigen capture ELISA is able to detect BVDV antigen in abomasal content of aborted fetuses. Due to transient viraemia, the antigen ELISA appears to be less useful for virus detection in acute BVD infections. In addition to above, our results showed that the antigen capture ELISA for detecting BVDV is technically time-consuming and labor-intensive than RT-PCR assay.

The previous study that comparison of RT-PCR with ELISA and cell culture immunoperoxidase tests for the detection of ruminant pestivirus infections revealed that RT-PCR is more sensitive than the other tests (Horner et al. 1995). The RT-PCR method can be adapted to the detection of BVD viral RNA for diagnostic purposes (Kim and obovi 2003, Letellier and Kerkhofs 2003). This may have a special value where low-level virus contamination is suspected, for example in biological products such as vaccines (sadeghi-nejad 1996).

Since, RT-PCR assay has been developed for the detection of BVDV in a wide variety of clinical samples such as formalin fixed paraffin embedded tissue sections (Gilbert et al. 1999), milk (Drew et al. 1999), blood (Hamel et al. 1995), follicular fluid (Kim and obovi 2003), Serum (Kosinova et al. 2007), skin samples (Passler et al. 2010), Ear notch (Kennedy et al. 2006) and in all of these studies RT-PCR has been introduced as an accurate and sensitive method to detection of BVDV. In these years many countries have accepted PCR as an accurate, fast and official assay to detection of various diseases such as BVD. Studies indicated that PCR is an accurate assay to detection of BVD's closely related disease such as Brucellosis (Buyukcangaz et al. 2011), Infectious Bovine Rhinotracheitis (IBR) (Takiuchi et al. 2005) and Leptospirosis (Richtzenhain et al. 2002) in clinical samples like aborted fetuses.

This present study showed that our RT-PCR test can be used as a commercial RT-PCR kit to detection of BVDV in various clinical samples.

In all RT-PCR assays it is important to adopt a nucleic acid extraction method which removes inhibitory factors and recovers most of the desired RNA.

BVD is a world-wide disease and reported from many sites of the world. The prevalence of BVDV in Bovine of Iran (20.48%) is higher than Argentina (1.69%) (Campero et al. 2003), Greek (14%) (Billinis et al. 2005) And India (17.31%%) (Sudharshana et al. 1999) But is lower than Northern Portugal (35%) (Niza-Ribeiro et al. 2005) and Turkey (23.07%) (Seyyal et al. 2002).

The previous study on Ovine aborted fetuses showed that placenta, heart, thymus and brain of fetuses are the most reliable tissues for BVDV antigen detection (Lamm et al. 2009).

To our knowledge there is no published data on prevalence of infected Bovine, Ovine, Caprine, Buffalo and Camel aborted fetuses with BVDV in Iran. So this study is the first report of direct identification of BVD virus by evaluation of antigen capture ELISA and RT-PCR in aborted Bovine, Ovine, Caprine, Buffalo and Camel fetuses in Iran.

Unfortunately, with all the precautions in this infection disease throughout the world it is not only still fully eradicated but also, it has a high prevalence in some areas like Iran (18.49% in this study). Aborted fetuses and reproductive discharge plays important roles in spread of disease and rapi, sensitive and trustful diagnostic methods can help to control disease.

The studies on Buffaloes and Camels are less than Bovine, Ovine and Caprine and this present study showed that the BVDV infection is one of the most prevalent causes of abortion in Buffaloes, Camels (in less amount) and as expected in Bovine. Disease is more prevalent in Caprine in contrast to Ovine. In the other hands, this present study showed that in addition to Bovine, BVDV can be a factor that cause abortion in Ovine, Caprine, Buffalo and Camel.

## Abbreviations

ELISA: enzyme linked immune sorbent assay; RT-PCR: reverse transcriptase polymerase chain reaction; BVDV: bovine viral diarrhea virus; ANOVA: analysis of variance; RNX: RNA extraction.

## Competing interests

The authors declare that they have no competing interests.

## Authors' contributions

All works such as collection of samples, RNA extraction, ELISA and RT-PCR performing, statistical analysis and writing of manuscript was done by Farhad Safarpoor Dehkordi.
